# Time Taken for Symptom Recognition, First Consultation, Diagnosis and First Definitive Treatment and Its Associated Factors among Women with Breast Cancer

**DOI:** 10.31557/APJCP.2021.22.11.3623

**Published:** 2021-11

**Authors:** Bachok Norsa’adah, Krishna Gopal Rampal, Rahmah Mohd Amin

**Affiliations:** 1 *Unit of Biostatistics and Research Methodology, School of Medical Sciences, Universiti Sains Malaysia, Kubang Kerian, Kelantan, Malaysia. *; 2 *Centre for Graduate Studies, Cyberjaya University College of Medical Sciences, Persiaran Bestari, Cyber 11, Cyberjaya, Selangor, Malaysia. *; 3 *Faculty of Medicine, Universiti Sultan Zainal Abidin, Medical Campus, Kuala Terengganu, Terengganu, Malaysia. *

**Keywords:** Breast cancer, treatment delay, patient delay, system delay

## Abstract

**Background:**

Breast cancer patients in Malaysia often present late, delaying diagnosis and treatment. Decisions on health-seeking behaviour are influenced by a complex interplay of several factors. Early detection and subsequent successful treatment are the main goal in order to reduce breast cancer mortality. The aims of this study were to identify the time taken by women with breast cancer for consultation, diagnosis and first definitive treatment and the factors associated with the initiation of definitive treatment.

**Methods:**

In this cohort study, we interviewed 328 women with histologically confirmed breast cancer at five medical centres in Malaysia. Times were measured from recognition of symptoms to first consultation to diagnosis and to the first definitive treatment. The event was initiation of definitive treatment. Data was analysed using multivariable Cox proportional hazards regression.

**Results:**

The mean age was 47.9 (standard deviation 9.4) years and 79.9% were ethnic Malays. The median follow-up time was 6.9 months. The median times for first doctor consultation, diagnosis and initiation of treatment were 2 months, 5.5 months and 2.4 weeks, respectively. The percentage of consultation delay more than a month was 66.8%, diagnosis delay more than three months was 73.2% and treatment delay more than one month was 11.6%. Factors associated with not initiating the definitive treatment were pregnancy (adjusted hazard ratio (AHR) 1.75; 95% Confidence Interval (CI): 1.07, 2.88), taking complementary alternative medicine (AHR 1.45; 95% CI: 1.15, 1.83), initial refusal of mastectomy (AHR 3.49; 95% CI: 2.38, 5.13) and undergoing lumpectomy prior to definitive treatment (AHR 1.62; 95% CI: 1.16, 2.28).

**Conclusions:**

Delays in diagnosis and consultation were more serious than treatment delays. Most respondents would accept treatment immediately after diagnosis. Respondents themselves were responsible for a large proportion of the delays. This study was successful in understanding the process of breast cancer patients’ experience, from symptoms recognition to consultation, diagnosis and treatment.

## Introduction

Breast cancer is the most common cancer in women worldwide and its incidence has increased over the years with industrialization and urbanization (Yen et al., 2016). There were 21,634 new breast cancers registered in Malaysia for the period of 2012-2016, compared to 18,206 cases reported in 2007-2011 (Azizah et al., 2019). The age-standardised incidence rates (ASR) of breast cancer in Malaysia has increased from 31.1 in 2007-2011 to 34.1 per 100,000 populations in 2012-2016 (Azizah et al., 2019). The ASR of breast cancer in Malaysia was not as high as in developed and western countries, but the majority of cases were at an advanced stage. The National Cancer Registry 2012-2016 reported that the percentage of people diagnosed with stage III and IV breast cancer were 25.1% and 22.8%, respectively, compared to the previous report 2007-2011 of 23.1% and 20.1% for stage III and IV, respectively (Azizah et al., 2019). 

Early detection of cancer is achievable and there are benefits to early stage diagnosis and treatment. The delay in presentation and detection of breast cancer patients is partially responsible for the advanced stage of presentation, and subsequently causes a delay in treatment and low survival rates (Caplan, 2014; Chukmaitov et al., 2018; Huo et al., 2015; Li et al., 2019a). The overall 5-year survival rate of breast cancer diagnosed in 2007-2011 in Malaysia was 61.9% (MySCan, 2018), which was low when compared to 90.3% in the United States in 2011-2017 (National Cancer Institute, 2021). A study of African American women reported that medical consultation delay more than 3 months was more likely to present with advanced stage of breast cancer (Gullatte et al., 2009). The 5-year survival in breast cancer patients with a treatment time of more than six weeks was 80% compared to 90% in those with treatment time less than two weeks (Smith et al., 2013). 

Delay is generally divided into patient delay, which is related to health seeking behaviour, and system delay, which is related to health care services (Caplan, 2014; Chukmaitov et al., 2018; Freitas and Weller, 2015; Rivera-Fronco and Leon-Rodriguez, 2018; Unger-Saldan et al., 2018). The patient delay was associated with advanced age (Chukmaitov et al., 2018), non-attribution of symptoms to cancer, fear of the disease and treatment and low educa¬tional level (Freitas and Weller, 2015). Women who delayed consultation usually underestimated their risk of developing breast cancer, while some women suffered fear, sadness, and worry regarding the diagnosis and possible treatments that influenced their decision to seek medical attention (Rivera-Fronco and Leon-Rodriguez, 2018). Failure of medical practitioners to refer or act appropriately, false negative mammograms and fine needle aspiration cytology (FNAC) results, were the main factors for system delay (Jenner et al., 2000). Factors associated with system delay were a young age and an unspecific presentation of breast symptoms (Rivera-Fronco and Leon-Rodriguez, 2018) and less comprehensive coverage of health insurance (Freitas and Weller, 2015).

Most research on breast cancer treatment delay has been done in developed countries, among minorities, while very few have been performed in less developed countries (Freitas and Weller, 2015). There has not been much published research in Malaysia. Research in this area is important for clinicians so they can better understand how to manage their patients, and for the Ministry of Health so that it can implement strategies and activities to prevent delays in the diagnosis and treatment of breast cancer. Most delays of disease management are preventable. Therefore, this study was conducted to identify the time interval from symptom recognition to first consultation, diagnosis and first treatment of breast cancer and the factors associated with treatment delay. 

## Materials and Methods

A cohort study was conducted in women newly diagnosed with primary invasive breast cancer by histopathological examination. We included only women with primary invasive breast cancers because their experience of detecting breast symptoms, getting it diagnosed and deciding whether to seek treatment were different from that of women with secondary or recurrent breast cancer. Respondents were from three referral medical centres in the East Coast of Malaysia and two major government hospitals in Kuala Lumpur. All respondents were recruited from in-patients and out-patients and selected by stratified sampling based on locality. We excluded people with cognitive problems and recurrent cancer. The respondents were met at diagnosis and retrospectively followed the history from first recognition of symptoms to the first healthcare consultation. From date of diagnosis, the respondents were prospectively followed until the initiation of first definitive treatment or when our study ended. Respondents were recruited in staggered depending on the walked in or admitted patients. The study was conducted in 12 months. Generally, data was collected at the first six months, with the followed-up progress over the next six months. The follow-up of each patient varied because they were not recruited at the same time. The median follow-up time was 6.9 months.

Face-to-face interviews were conducted using structured questionnaires to standardise data collection, as some of our respondents were illiterate. The questionnaires comprised socio-demographic data, medical and obstetric history, clinical presentation, use of complementary alternative medicine (CAM), diagnostic tests and treatments. Information from medical records was also extracted, such as referral details, clinical presentations, diagnostic tests, histopathological reports and treatments. Dates of all the chronological events of breast cancer were collected; including the first recognition of symptoms, first consultation, referral, first hospital appointment, first meeting with surgeon and oncologist, diagnostic testing, surgical management, and first chemotherapy and radiotherapy. The time was recorded from the date of recognition of symptoms until the first consultation, the final diagnosis by histopathological examination and the beginning of treatment. If the respondent did not detect any symptoms herself, the date of abnormal clinical breast examination or date of screening mammogram was taken (Jaiswal et al., 2018). Delay was defined based on the time interval taken by the women. Consultation delay was defined as a time of more than one month from the recognition of symptoms to the first medical consultation (Harirchi et al., 2015). Diagnosis delay was defined as a time from symptom recognition to final diagnosis of more than three months (McGee et al., 2013). Treatment delay was defined as a time taken from histopathological diagnosis to initiation of treatment, either surgical or systemic, of more than one month (McGee et al., 2013; Mohd Mujar et al., 2017; Shandiz et al., 2016). Systemic treatment refers to any cancer treatment given to the breast cancer patients that is given intravenously or orally, such as chemotherapy. 

Patients delay was considered if the patient intentionally delayed medical care due to a lack of knowledge, assumption that the symptoms were due to other diseases, desire to wait for either symptom progression or disappearance, taking CAM, lack of time due to competing priorities, the patient was outside of the area or the patient defaulted on the appointment (Caplan, 2014). System delay occurred within the health care system included failure of FNAC to detect enough cells, benign reporting on FNAC, negative mammograms, failure of medical practitioners to refer or instruct appropriate diagnostic tests and clinical errors, such as failure to palpate the mass or assigning appointments (Barber et al., 2004). 

Co-morbidity is defined as any other diagnosed diseases of the respondent. CAM is defined as any usage of methods, practices or products for the purposes of medical and health care that are not part of the conventional modern medicine. We considered the patients as pregnant if their breast cancer was diagnosed during their pregnancy or either before or during their treatment. Respondents were considered as refusing treatment if they had not yet begun therapy and had no intention of taking any recommended treatment by the end of the study. 

*Statistical Analyses*

Data was analysed using SPSS version 26. Continuous data was summarised as mean (standard deviation (SD)) or median (interquartile range (IQR)), depending on the normality of distribution, while categorical data were presented as frequency (percentage (%)). Delay rates were summarised as frequency and percentage. 

Multivariable survival analysis, Cox proportional hazards regression model was used to determine predictors for no initiation of definitive treatment. Treatment time is measured in days from the date of the histopathology report of the breast tissue until the date of the first definitive treatment. For respondents who had not received any definitive treatment, their date of treatment was the end of study. The event was coded as one, which included respondents who received definitive treatment of either surgery or chemotherapy or radiotherapy, and the censor was coded as zero, which included respondents who had not received definitive treatment. A unique feature of survival analysis is that not all respondents experience the event at the end of the observation period, this is called censor (Schober and Vetter, 2018). In this study, not all respondents agreed to initiate definitive treatment for breast cancer. A stepwise selection was used with automatic insertion of any variable with p value <0.05 and deletion of any variable with p value > 0.1, one at a time. Results of the analysis are presented as crude and adjusted hazard ratio (HR), 95% confidence interval (CI) and p value. A hazard ratio greater than one indicates a high probability of refusing or delaying a treatment, while a hazard ratio less than one indicates higher probability of starting treatment. The significance level is determined as the value of p <0.05. 

## Results

We included 328 respondents with a median follow up time of 6.9 months from the date of first recognition of symptoms until the end of the study. Table 1 presents the socio-demographic profiles of the respondents with a mean age of 47.9 (SD 9.4) years. Most of the breast cancer patients were above 40 years old (82.9%), ethnic Malays (79.9%), had at least a high school education (39.0%), were married (79.3%) and were housewives (54.9%). Table 2 shows the medical history and clinical presentations of the respondents. Only 18.9% of the respondents had a family history of breast cancer, while 31.4% had concurrent diseases, 12% had no children and 30.5% were post-menopausal. Breast lump was the most frequent first symptom (87.8%). Eighteen respondents were pregnant at diagnosis or became pregnant prior to starting or during treatment. Fourteen of the 15 patients who were pregnant at diagnosis delayed diagnosis for three months or more. Eleven patients began treatment within a month of diagnosis and three patients began treatment after three months. 

The majority of breast cancers were invasive ductal carcinoma, as shown in Table 3. Grade 2 breast cancer was identified in 45.7% of the patients, while 44.8% had stage III and 11.3% had stage IV disease. The majority underwent mastectomy (73.8%) with completed chemotherapy (86.3%) and radiotherapy (78.4%). At the diagnosis, 48 (14.6%) breast cancer patients refused mastectomy. By the end of the study, only five patients had refused all treatments. Of the 18 inoperable patients, 16 completed chemotherapy and four completed radiotherapy while two did not undergo any treatment. Therefore, a total of seven patients did not receive any treatment by the end of our follow-up. 

*Delay in Breast Cancer Patients*

Table 4 shows the time taken from initial symptom recognition until consultation, diagnosis and treatment initiation. The overall range for recognition of symptoms until initiation of treatment was 0.3 to 16 years with a median of 206.5 days, until consultation time was 0 to 11 years with a median of 62 days and until diagnosis time was 2 to 16 years with a median of 167.5 days. The range of treatment time was 0 to 2.8 years and the median was 17 days. Approximately 43.3% of respondents delayed the consultation by more than three months, 73.2% delayed the diagnosis time by more than three months and 27.4% delayed the treatment time by more than a month. 

Table 5 shows the causes of diagnosis and treatment delays. The majority of the breast cancer patients received delayed the diagnosis (55.8%) and treatment (19.8%) due to patient delay; many of the patients sought alternative treatment rather than going to the hospital. Meanwhile, problems related to doctors and appointments, FNAC and mammograms contributed to system delay, specifically 12.5% for diagnosis and 3.7% for treatment. 

Predictors for not initiating the first definitive treatment are shown in Table 6. Significant factors were pregnancy (adjusted hazard ratio (AHR) 1.75; 95% Confidence Interval (CI): 1.07, 2.88), taking complementary alternative medicine (AHR 1.45; 95% CI: 1.15, 1.83), initial refusal of mastectomy (AHR 3.49; 95% CI: 2.38, 5.13) and undergoing lumpectomy prior to definitive treatment (AHR 1.62; 95% CI: 1.16, 2.28). 

**Table 1:** Socio-Demographic Characteristics of the Respondents (n=328)

Socio-demography	Frequency (%)	Mean (SD)
Age at diagnosis (year)		47.9 (9.4)
40 & less	56 (17.1)	
> 40	272 (82.9)	
Ethnicity		
Malay	262 (79.9)	
Non-Malay	66 (20.1)	
Education level		
No formal education	39 (11.9)	
Primary	52 (15.9)	
Secondary	178 (54.2)	
Tertiary	59 (18.0)	
Marital status		
Married	260 (79.3)	
Widow	39 (11.8)	
Single	18 (5.5)	
Divorce	11 (3.4)	
Occupation		
Housewife	180 (54.9)	
Government servant	78 (23.8)	
Private sector	44 (13.3)	
Self-employed	13 (4.0)	
Unemployed	13 (4.0)	
Monthly family income (MYR)		1500 (2338)*

*median (IQR), US$1, Malaysian Ringgit (MYR) 0.25

**Table 2:** Medical History and Clinical Presentations of the Respondents (n=328)

Medical History	Frequency (%)
Family history	
First degree relative	27 (8.2)
Distant relative	35 (10.7)
None	266 (81.1)
Co-morbidity	103 (31.4)
Parity status	
Nulliparous	40 (12.2)
Parous	288 (87.8)
Menopausal status	
Pre	228 (69.5)
Post	100 (30.5)
Pregnant at or post diagnosis	18 (5.5)
First symptom	
Lump at breast	288 (87.8)
Nipple problems	12 (3.6)
Pain	10 (3.1)
Change of breast shape	9 (2.8)
No symptom	4 (1.2)
Others	5 (1.5)

**Table 3:** Histopathological Findings and Treatment of the Respondents (n=328)

	Frequency (%)
Histopathology types	
Invasive ductal	293 (89.3)
Invasive lobular	13 (4.0)
Mucinous	6 (1.8)
Medullary	4 (1.2)
Others	12 (3.7)
Bloom Richardson Grade	
1	55 (16.8)
2	150 (45.7)
3	123 (37.5)
Stage of cancer	
I	17 (5.2)
II	127 (38.7)
III	147 (44.8)
IV	37 (11.3)
Type of surgery	
Mastectomy	242 (73.8)
Lumpectomy/quadrectomy	63 (19.2)
Lumpectomy before mastectomy	39 (11.9)
Refused surgery	5 (1.5)
Inoperable	18 (5.5)
Breast reconstructive (Yes)	24 (7.3)
Chemotherapy	
Completed	283 (86.3)
Not completed	9 (2.7)
Not suggested	29 (8.8)
Refused	7 (2.2)
Radiotherapy	
Completed	257 (78.4)
Not completed	2 (0.6)
Not suggested	65 (19.8)
Refused	4 (1.2)
Complementary alternative medicine	
Yes	140 (42.7)
Tamoxifen	
Yes	180 (54.9)

**Table 4:** Consultation, Diagnosis and Treatment Delay and Percentage of Delay in Breast Cancer Patients (n=328)

Types of delay	Range (years)	Median (days)	Mean (days)	Month(s)	Frequency (%)
Symptom recognition to consultation	0-11.0	61	216		
				0-1	109 (33.2)
				>1-2	44 (13.4)
				>2-3	33 (10.1)
				>3	142 (43.3)
Symptom to surgeon / oncologist	0-15.9	158.5	360.8		
Symptom to diagnosis	2-16.0	167.5	374		
				0-3	88 (26.8)
				>3	240 (73.2)
Diagnosis to first treatment	0-2.8	17	54.7		
				0-1	328 (72.6)
				>1	90 (27.4)
Symptom to treatment	0.3-16.0	206.5	428.6		

**Table 5:** Causes of Diagnosis and Treatment Delays in Breast Cancer Patients (n=328)

	Frequency (%)
1. Diagnosis delay due to Patient Delay	183 (55.8)
Complementary alternative treatment	116 (35.4)
Defaulted appointment	48 (14.6)
Did not take symptom seriously	35 (10.7)
Attribute symptom as benign disease	30 (9.1)
Fear	25 (7.2)
Pregnant	14 (4.3)
Other priority	10 (3.0)
Family sanction	6 (1.8)
Denial	3 (0.9)
2. Diagnosis delay due to System Delay	41 (12.5)
Negative FNAC	31 (9.5)
Inadequate FNAC	30 (9.1)
Doctor inaction	30 (9.1)
Doctor told not cancer without biopsy	19 (5.8)
Negative mammogram	13 (4.0)
3. Diagnosis delay due to Patient & System Delays	16 (4.9)
4. Treatment delay due to Patient Delay	65 (19.8)
Complementary alternative treatment	57 (17.4)
Refused mastectomy	32 (9.8)
Defaulted appointment	29 (8.8)
Fear	8 (2.4)
Refused chemotherapy	6 (1.8)
Could not accept diagnosis	6 (1.8)
Pregnant	4 (1.2)
Family sanction	4 (1.2)
Wanted to wait	2 (0.6)
Financial	1 (0.3)
Not ready	1 (0.3)
Busy	1 (0.3)
	Frequency (%)
5. Treatment delay due to System Delay	12 (3.7)
Already had done lumpectomy	18 (5.5)
Inoperable	8 (2.4)
Waiting result	5 (1.5)
6. Treatment delay due to Patient & System Delays	13 (4.0)

Diagnosis delay is defined as time from recognition of symptom to diagnosis >3 months; Treatment delay is defined as time from diagnosis to first treatment >1 month; Some participants may have had more than one reasons.

**Table 6:** Predictors for Non-Initiation of First Definitive Treatment in Women with Breast Cancer (n=328)

Predictor factors	Crude Hazard Ratio^a^ (95% CI)	P value^a^	Adjusted Hazard Ratio^b^ (95% CI)	P value^b^
Pregnant	1.98 (1.21, 3.24)	0.007	1.75 (1.07, 2.88)	0.027
Complementary alternative medicine	1.87 (1.49, 2.36)	<0.001	1.45 (1.15, 1.83)	0.002
Initial refusal of mastectomy	3.91 (2.70, 5.65)	<0.001	3.49 (2.38, 5.13)	<0.001
Lumpectomy before mastectomy	1.24 (0.89, 1.74)	0.208	1.62 (1.16, 2.28)	0.005

^a^Univariate Cox Proportional Hazard; ^b^Multiple Cox Proportional Hazard

## Discussion

The most common presentation of our respondents was breast lump, as supported by other studies (Harirchi et al., 2015; Li et al., 2019b). Most breast cancer in Malaysia is detected by the patients themselves; mammogram is not generally utilised in detection in asymptomatic patients like it is in the developed countries, signifying an inadequacy in early screening for breast cancer. Our study found five breast cancer patients who refused any recommended treatment and two patients who were inoperable and not offered any specific treatment by the end of study (2.1%). Refusal of medical treatment happened in Malaysia; a report in Sabah found 20.4% of patients refused treatment (Leong et al., 2007).

*Time interval and proportion of presentation, diagnosis and treatment delays*

Methodological issues exist related to measuring delay and the inconsistent specifications of delay types (Andersen et al., 2009). There are many different definitions of delays and ways of categorising the delays (Freitas and Weller, 2015). There is no consensus on the time frame that constitutes a delay (Jaiswal et al., 2018). Some studies have measured delay by using median cut off points (Jaiswal et al., 2018), or a time from the initial symptom to breast cancer diagnosis of more than 30 days (Huo et al., 2015). Other studies have considered patient delay as a period of at least 3 months from either the first sign or symptom to seeking medical attention (Jenner et al., 2000; Li et al., 2019b), and care delay as a period of at least 1 month from seeking medical attention to receiving therapy (Li et al., 2019b). 

Our study found that the median time between discovery of breast symptoms to consultation was 62 days, which was similar to the result reported in another Malaysian study of 2.4 months (Mohd Mujur et al., 2017). In comparison, an Indian study reported a presentation delay of 35 days (Kumar et al., 2019) while, in China, 2 months has been reported (Li et al., 2019b). Approximately 43.3% of our respondents delayed the consultation by more than one month. In Iran, 31.7% of patients took longer than one month from first detecting symptoms to visit a health care provider (Harirchi et al., 2015) while this was only 15.5% in China (Li et al., 2019b). Other studies have reported 20-40% of breast cancer patients taking greater than 3 months to visit a provider (Li et al., 2019b; Khan et al., 2015; Mohd Mujur et al., 2017; Unger-Saldaña et al., 2018).

The median time to diagnosis in our study was 167.5 days while, in 73.2% of cases, delayed the diagnosis time by more than three months. These findings were very poor in comparison to similar results of 26 days between presentation and diagnosis in another Malaysian study (Mohd Mujar et al., 2017), 23 days in breast cancer patients of a hospital-based cancer registry in Denver (Jaiswal et al., 2018) and 31 days in Portugal (Nouws et al., 2019). Only 17.9% of breast cancer patients in Iran had more than one week between the first medical visit for the symptoms and the diagnosis (Harirchi et al., 2015). Diagnosis intervals greater than 3 months were reported in 65% of participants in a study in Mexico (Unger-Saldaña et al., 2018). A study in Malaysia found a delay in diagnosis time of more than 1 month, taken from the first presentation at a primary care facility until definitive diagnosis, was 41.8% (Mohd Mujar et al., 2017). 

The median treatment time of our study was 17 days and 27.4% delayed the treatment time by more than a month. In comparison, another Malaysia study found a median treatment time of 24.7 days while 35.3% of the patients started treatment more than one month after their diagnosis (Mohd Mujar et al., 2017). The median times between first detection and first treatment were 44 days in a study of Portugal (Nouws et al., 2019) and 37 days in a study of Denver (Jaiswal et al., 2018), while the median time from diagnosis until treatment was 27.0 days in a study in North Carolina (McGee et al., 2013) and a very long 130 days in a study in India (Kumar et al., 2019). A large study from the US National Cancer Database reported a median time from biopsy to first treatment of 35.6 and 33.4 days for neoadjuvant and adjuvant chemotherapy patients (Melchior et al., 2020). Treatment delay for more than one month was found in 10.7% of patients in a study in Kazakhstan (Chukmaitov et al., 2018), 28.3% in a study in Iran (Harirchi et al., 2015) and 39.5% in a study in North Carolina (McGee et al., 2013). Also, our study found that the median overall time between recognition of symptoms and initiation of treatment was 206.5 days, which is similar to a study in India that found 203 days (Kumar et al., 2019). In comparison, the median time of total delay was 95 days in Guangzhou (Li et al., 2019b). The proportion of treatment delay in our study was either lower than or comparable to those found in other studies in developed countries; this is probably related to the fact that cancer treatment is subsidised by the government and readily available to the population.

*Causes of delays*

Patient delay was more common than system delay or care delay. The patient delay contributed to 55.8% of the diagnosis and 19.8% of the treatment delays in our study. Many respondents seek alternative treatment rather than seek treatment from hospitals. Other causes of delay included defaulting on appointments, taking the symptoms lightly or as indicating benign diseases, fear of treatment and pregnancy. Meanwhile, problems related to FNAC, mammograms and failures by doctors to take actions contributed to the system delay. A review of the literature shows that the most frequent causes of patient delay are non-attribution of symptoms to cancer, fear of the disease and treatment and low educational level. Meanwhile, less comprehensive health insurance coverage, older/younger age and false negative diagnostic tests were the three most common causal factors of system delay (Freitas and Weller, 2015).

A study in China reported 40.4% patient delay and 15.5% care delay (Li et al., 2019b). In Iran, the main reasons for treatment initiation greater than one week from confirmed diagnosis of malignancy were long waiting lists to receive treatment (49.5%), lack of availability of treatment facilities (14.7%) and financial problems (11%) (Harirchi et al., 2015). Among 72 UK breast cancer patients with delayed diagnosis, only three patients (4.2%) delayed due to the patient’s own reasons (Barber et al., 2004). Another study found that, out of 42 cases of delay in diagnosis of three months or more, only one patient (2.4%) did not follow the doctor’s advice, the other cases of delay were due to the medical practitioner or health-care system (Jenner et al., 2000). These results are in contrasted to our study, probably due to patients in developed countries being more knowledgeable and health care systems that rely on health insurance and the patients’ own money for treatment. 

*Factors associated with non-initiation of definitive treatment*

Our study found that treatment delay was significantly associated with pregnancy, usage of CAM, initial refusal of mastectomy and undergoing lumpectomy prior to definitive treatment, which were related to patient and system delays. The literature shows numerous factors inconsistently associated with treatment delay. 

The percentage of breast cancer during pregnancy in our study was 5.5% because our respondents were relatively younger, pre-menopause women. The Dutch Pathology Registry reported 6.9% of pregnancy-associated breast cancer in women younger than 45 years (Suelmann et al., 2021). The breast cancers in pregnant women were found to be more aggressive in histopathologic profile than the non-pregnant controls. Diagnosis and treatment delays of breast cancer frequently occurred in pregnant women. Delay in diagnosis during pregnancy occurs indirectly related to the fact that most patients are relatively younger, the symptom is usually blamed on the influences of pregnancy-related hormones on the breast, and decreased diagnostic testing during pregnancy by medical practitioners (Suelmann et al., 2021). A retrospective study in Texas reported that 25 of 51 breast cancer patients did not received treatment during pregnancy. The study also found a significantly more advanced TNM classification in pregnant women with breast cancer compared to those who were not pregnant (Beadle et al., 2009). 

Our study found that the use of CAM was significantly associated with delay of treatment. However, a multicentre study in Malaysia found that the usage of CAM was significantly associated with presentation and diagnosis delays but not treatment delay (Mohd Mujar et al., 2017). In our study, 42.7% of respondents took CAM, which was similar with the 46.5% found in the previously mentioned study (Mohd Mujar et al., 2017), but lower compared to a report in Germany, which found 62.5% of young breast cancer patients used CAM (Hammersen et al., 2020). A study in Northern Pakistan reported that 40.7% of patients who delayed more than 3 months took some time to use alternative medicine (Khan et al, 2015). In a study conducted among the poor, non-educated rural patients in East Malaysia, 20.4% defaulted on treatments and most opted for traditional alternatives (Leong at al., 2007). Regarding CAM usage in Malaysia, 75.9% of breast cancer patients used biological based CAMs and the usage was significantly associated with Malay ethnicity, advanced stages of disease, non-adherence to treatments and if the patients interpreted their symptoms as not related to cancer (Mohd Mujar et al., 2017). CAM use was significantly higher in women with a higher educational level and better employment status (Hammersen et al., 2020). Most women with advanced stages of cancer choose alternative treatments, only opting for hospital treatment when all other efforts have failed and the symptoms become more serious (Taib et al., 2007). Traditional Malay healers embedded in the Malay culture may involve shamans, who use traditional methods in diagnosing and treating patients, including herbal remedies, ceremonial rites, incantation, exorcism and sorcery (Razali and Najib, 2000). Most patients in our study had taken chanted water or herbal local application at the breast lump. Many breast cancer patients also took dietary or nutritional supplements and practiced spiritual alternative treatments (Mohd Mujar et al., 2017, Zulkipli et al., 2018). 

Some patients prefer alternative treatments for various reasons, including the fact that it has been recommended by family, friends or other survivors and perceived to be effective with fewer side effects (Zulkipli et al., 2018). Alternative treatments may pose a hazard risk as the effectiveness of these various methods are unknown, while completely abandoning conventional treatments may cause the cancer to spread. It was found that 43% of cancer patients who practiced complementary treatment did not tell their doctor (Yates et al., 2005). Another study found that every fifth woman used CAM without her physician’s knowledge (Hammersen et al., 2020). 

When mastectomy was first advised, 14.6% of our respondents refused. There were fears related to the treatments and prognosis of breast cancer, as reported among South Africans, particularly the adverse effects of chemotherapy, radiation, hair loss, and loss of the breast (Rayne et al., 2016). The breast is an organ that identifies the gender of a woman and contributes to her body image (Kocan and Gursoy, 2016). Furthermore, it is linked to femininity, beauty and motherhood. The suggestion for mastectomy is associated with abundant stress, fear for loss of femininity and self-identity and a declining spousal relationship (Olasehinde et al., 2019). Some women may decide not to get the appropriate treatment over fear of husband abandonment, him taking another wife or their marriage ending with divorce (Sanchuli et al, 2017). Furthermore, there are women who worry about losing their ability to work again after mastectomy and thus being dependent upon others (Burgess et al., 2006). Some women worry that they may lose their profession (Rayne et al., 2016) or be unable to perform their routine and daily chores and that it will disrupt their social relationships (Kocan and Gursoy, 2016). 

Of our respondents, 11.9% either had a lumpectomy with a diagnosis of benign breast disease that histopathologic examination later confirmed to be breast cancer or were respondents who were suggested for breast conservation surgery but histopathologic examination reported remaining cancer cells at the periphery of the surgical site, leading to a second surgery; these patients had subsequent definitive mastectomy done. Any prior procedure would delay definitive treatment, including breast reconstruction. Lumpectomy should not be used to biopsy breast cancer. If breast cancer has been confirmed, surgery with an adequate periphery should be performed, as a surgical site that is too close to the cancer cells will cause cancer to relapse quickly (Corsi et al, 2013). A review article reported that 1.7-3.2% of local recurrences for surgical margins of 5 mm compared to 10.5-16% of local recurrences for surgical margins of 1 mm (Corsi et al., 2013). Furthermore, some patients were reluctant to undergo surgery for the second time.

There were many other factors inconsistently associated with treatment delay reported in the literature. Factors associated with treatment delay included low socioeconomic status (Chavez-MacGregor et al., 2016; Harirchi et al., 2015), Hispanic ethnicity or non-Hispanic black race (Chavez-MacGregor et al., 2016; Melchior et al., 2020), educational level (Harirchi et al., 2015; Li et al., 2019a; Kumar et al., 2019), premenopausal status, history of benign breast disease and less physical examination (Li et al., 2019b), breast reconstruction (Chavez-MacGregor et al., 2016) and non-private insurance (Chavez-MacGregor et al., 2016). All of these factors are not significant in our study because the majority of our respondents have a low socio-economic status and treatment cost is not a major issue.

A major strength of the study was this was a multi-centre study involving five large medical centres. The selected medical centres were large general and university hospitals that serve a lower a socio-economic population. Chances of breast cancer patients having treatment elsewhere were minimal because cancer treatment is expensive and most patients could not afford private services. We believed that our data is reliable. The times were calculated by collecting dates from patients with support from medical records when they were newly diagnosed, thus minimising recall bias. We clearly identified the stages that the women with breast cancer encountered, starting with the recognition of symptoms and including first presentation to a health care professional, subsequent diagnosis and initiation of treatment, as defined by the Andersen Model (Walter et al., 2012). Our study interviewed respondents face-to-face by using standardised questionnaires, this led to complete response rates compared to had the study used medical records, postal or telephone interviews. Missing data were avoided and variables were collected with standardised definitions. We thought that this study was comprehensive since it covered all important factors of delay. 

This study has several limitations. We were using convenient sampling that might not be representative of the Malaysian population. The study was conducted prospectively by interviewing respondents at diagnosis; thus, this study did not include patients who had died or those who did not receive appropriate care for breast cancer. We presumed those who had died were in advanced stages of breast cancer and took more time for treatment. Social desirability bias could occur as the patients might respond favourably to questions related to their delay. Selection bias might also have occurred because this study was conducted in hospitals and some patients who prefer alternative treatment may not come to a hospital. Population-based study was not possible because of logistic problems in finding those patients. Moreover, there was information bias. We relied on the patients’ recall of the events leading up to their diagnosis and treatment. Patients who delayed needed to remember the events more retrospectively than those who did not delay. 

In conclusion, this study was conducted in five medical centres in Malaysia among a low socio-economic population. More than half of respondents were in advanced stages of disease. Consultation and diagnosis delays were very serious in this study compared to treatment delay. Most of the delays were due to patient delay. The significant predictor factors for treatment delay were pregnancy, intake of CAM, refused mastectomy and undergoing lumpectomy prior to definitive treatment. 

Prevention should be taken to minimise the time taken for diagnosis and treatment through regular breast cancer awareness and education promoting early detection. Patients need role models of breast cancer survivors to avoid negative perceptions about the treatment of breast cancer. Non-governmental organisations, welfare departments and social support volunteers can provide support for patients who have difficulty accepting treatment. Efforts should focus on strengthening the quality of primary care services and improving referral and treatment pathways to cancer care services. Doctor-patient communication has to be effective so that patients can accept the doctors’ suggestions for treatment. Clinical practice guidelines for breast cancer management should incorporate time guidelines for diagnosis and treatment. This also serves as a quality assessment of services by the Ministry of Health. All breast cancer patients should receive timely diagnosis and treatment.

## Author Contribution Statement

The authors confirm contribution to the paper as follows: Conception and design of the study: BN. Collection and acquisition, analysis and interpretation of the data: BN. Obtaining of funding: BN, KGR Administrative, technical, or logistic support: BN, MAR, KGR Drafting of the article: BN. Critical revision of the article for important intellectual content: BN, MAR, KGR. All authors approved the final version of the paper.

## Funding statement

This study was funded by USM short-term grant 304/PPSP/6131559 and UKM Fundamental Fund FF-130-2007. 

## Ethical Approval

This research had been approved by the Research and Ethical Committees from all the respective institutions, with the reference numbers: UKM 1.5.3.5/244/SPP 2, HKL/98/AM 882, USMKK/PKK/JK EP(M) 191 USM, Bil (43), HRPZ ll.71/20 Jld.8, HSNZ.KT.100-22/15(27) and KKM/NIHSEC/08/0804/P07-13. All respondents signed a consent form to participate in the study. All information was kept confidential.

## Data availability statement

All data collected in in this study are in the guardianship of the researchers and will be available upon request.

## Conflict of interest statement

There is no conflict of interest.
